# Methodological challenges in neonatal microbiome research

**DOI:** 10.1080/19490976.2023.2183687

**Published:** 2023-02-26

**Authors:** Jonathan A Chapman, Christopher J Stewart

**Affiliations:** Translational and Clinical Research Institute, Newcastle University, Newcastle upon Tyne, UK

**Keywords:** Neonatal gut microbiome;, neonatal health;, gut microbiota composition;, gut microbiome development;, microbiome analytical methods;, neonatal models;, intestinal organoids;, host microbiome interactions;, study design

## Abstract

Following microbial colonization at birth, the gut microbiome plays a vital role in the healthy development of human neonates and impacts both health and disease in later life. Understanding the development of the neonatal gut microbiome and how it interacts with the neonatal host are therefore important areas of study. However, research within this field must address a range of specific challenges that impact the design and implementation of research methods. If not considered ahead of time, these challenges have the potential to introduce biases into studies, negatively affecting the relevance, reproducibility, and impact of any findings. This review outlines the nature of these challenges and points to current and future solutions, as outlined in the literature, to assist researchers in the early stages of study design.

## Introduction

The human gut is colonized by a vast array of microbes, which form a highly complex and diverse community comprised of bacteria, fungi, viruses and archaea.^1^ This microbiome has been demonstrated to play vital roles in numerous physiological processes within the host, including but not limited to: development and regulation of the immune system, alterations in neurological signaling, biosynthesis of vitamins and amino acids, digestion of food and suppression of pathogen growth. The gut microbiome is therefore of fundamental importance to the development and maintenance of normal human health. Abnormal development of the gut microbiome can accordingly have negative impacts on the health of the host. Disruption of gut microbiota community composition has been linked to gastrointestinal, neurological, metabolic, hepatic, autoimmune, cardiovascular and oncologic diseases.^2,3^ This prominence in both health and disease is especially true for neonates, where both the microbial community and the host immunity and barrier defenses are developing. Microbial colonization of the gut generally begins at birth, with exposure of the infant to viable microbes during exit from the womb.^4^ The gut microbiome is then critical within the first year of life for training the infant’s immune system and promoting long-term healthy physiological development. Abnormal gut microbiome development during the earliest stages of life is associated with severe pathologies shortly after birth, such as necrotizing enterocolitis (NEC), an inflammatory disorder of the bowl that is one of the leading causes of mortality amongst preterm infants.^4,5^

Gaining mechanistic understanding of neonatal gut microbiome development and how the microbiome and the host interact is therefore of vital importance for maintaining and improving health outcomes within our populations that can persist into childhood and beyond. Importantly, research in this field must address an array of challenges, which can be broadly grouped into 3 areas:

1. Design and recruitment of representative cohorts for studies
2. Accurate detection of the composition of the gut microbiome
3. Studying interactions between the neonatal host and the gut microbiome

This review aims to outline the nature of these challenges, particularly in neonatal research, and provide relevant information to inform investigators at the early stages of initiating a study.

## Design and recruitment of representative study cohorts

Despite being less diverse than that of adults, the neonatal gut microbiome remains a complex system that is partly shaped by genetic and environmental factors and therefore has emergent properties which differ between infants.^6,7^ As such, there are a variety of covariates and confounding factors that will impact study results. These can include an infant being born prematurely, diet (especially receiving breastmilk), living in a household with furry pets, use of probiotics, antibiotic treatments, rural or urban residence, frequency of specific alleles, ethnicity and parental socioeconomic status.^6–9^ Maternal health factors must also be considered when analyzing study data, as use of antibiotics, maternal BMI, dietary intake and occurrence of gestational diabetes have all been shown to impact neonatal microbiome development.^10,11^ Cohorts for studies must therefore be carefully designed so as to reflect the balance of covariates within the population of interest and extricate confounders where possible. This has been eloquently demonstrated in adult populations, where matching a range of high-burden human diseases with controls for confounding variables reduced both the number and strength of statistical significance of associations between cases and controls.^12^

### i) Cohort design

Cohort size is an important factor in this regard and is particularly key for determining the statistical power of a trial.^13^ Given the numerous approaches to analyzing a microbiome dataset (e.g., alpha diversity, beta diversity, relative taxonomic abundance), performing power calculations is not trivial and can be subjective (this is reviewed in detail elsewhere).^14,15^ The smaller the effect size of a variable, the larger the sample size needed to detect the relevant signal and overcome masking by confounders.^13,16^ This is especially true when studying the effect of host genetics on the microbiome. Direct sequencing of candidate genomes to identify alleles of interest would require hundreds of neonates, while genome wide association studies (GWAS) usually require cohorts numbering in the thousands.^9,13^ However, recruiting a large enough sample size can be challenging when addressing research questions focused on neonatal microbiome phenotypes and related pathologies. A key example of this is the study of NEC in preterm infants. Only 10% of babies are born preterm and, of those, roughly 5–10% will suffer from NEC.^9^ This leaves researchers with a limited patient pool from which to draw on for studies.

Conducting a multi-center study, ranging across either an entire country or international borders, provides a way to maximize sample size when patient numbers are limited. Recruiting from multiple geographic locations will also likely increase the demographic diversity of the cohort, making it more representative and ultimately more generalizable. Beyond observational studies, embedding microbiome research into randomized controlled trials (RCTs) can allow potential mechanisms to be explored. For instance, where the invention is found to be effective in patients with a particular baseline microbial community, or where the efficacy is dependent on modulation of the infant microbiome. An example of this is the Mechanisms Affecting the Gut of Preterm Infants in Enteral feeding (MAGPIE) study, conducted in the UK, which enrolled 479 preterm infants from 12 NHS hospitals.^17,18^ Of course, large cohort, multi-center studies do complicate the logistics of sample collection, requiring samples to be stored and then transported to a central location for analysis. Sample storage methods and their impacts on microbiome integrity will be discussed in the next section. Furthermore, larger and more diverse cohorts may introduce additional confounders that would be absent when using a smaller and more uniform cohort. It should be noted that regardless of the size or geographic distribution of a cohort, any findings will likely be restricted to the local, national or continental populations surveyed in the study. The large number of covariates and confounders that impact the gut microbiome makes results hard to extrapolate to all neonates, especially when considering industrialized vs. non-industrialized nations. Should the biological mechanisms underlying observed associations be fully understood, then wider extrapolation beyond narrow population groups could possible, due to the fundamental similarities in biology shared by all humans.

### ii) Additional methods to control for confounders

In addition to optimizing cohort size and geographic distribution, there are methods that can be used to address the confounders that microbiome studies are particularly susceptible to. Restriction is a simple tool whereby the cohort is limited based on a set of characteristics, so as to reduce confounding by those characteristics.^19^ For instance, this might include restricting a study to include only neonates born after 37 weeks’ gestation, which should remove confounding microbiome effects by being born preterm vs term. If a particular phenotype or pathology is being studied, then matching can be deployed, whereby phenotype/pathology positive and negative groups can be matched in pairs based on potential confounders.^19^ This has been used in NEC studies, where NEC and non-NEC neonates were matched by various traits, including gestational age, birthweight and whether they received probiotics.^20^ Both restriction and matching, along with sample size and geographic location, form part of initial cohort design. There are also analytical approaches for protecting against confounders after a dataset has already been collected, namely stratification and multivariate analysis.^19,21^

Stratification involves dividing the study cohort into strata based on potential confounders, such as age or sex.^19,21^ Associations within specific strata that differ from those seen across the entire cohort can then be observed, thus confirming whether a variable is a confounder or not.^21^ For cohorts with a large number of metadata categories however, stratification could be too complex and time consuming, as the number of strata to analyze rapidly increases with each additional variable. Multivariate analysis is better suited for such datasets. Variables of interest are investigated and potential confounding variables are adjusted for, using one integrated mathematical process. In addition, stratification can reveal effects that are associated with specific sub-strata that could be missed with multivariate analysis and adjustment. A further advantage of stratification is that interaction effects can be more easily identified within sub-strata. An interaction effect is the joint effect of two or more variables upon the dependent variable, in this case gut microbiome composition, which is greater than their individual effects. Stratifying data by, for example, whether an infant was breast fed, would allow for clearer observations of interaction effects between breastfeeding and other variables and their impacts on gut microbiome composition.

As microbiome datasets are generally highly multi-dimensional, these analytical approaches are essential for generating meaningful data from studies, while careful design to ensure cohorts are large enough and representative enhances the relevance of any findings.

## Accurate detection of the composition of the neonatal gut microbiome

Accurate analysis of the gut microbiome to detect associations with covariates is dependent upon accurate detection of microbiota composition. Entire studies are premised on this process being robust and precise. But it has been shown that experimental design and choice of methods can affect stool microbiota composition and bias downstream analysis.^22,23^ This is the case for all stages of sample preparation, incorporating sample collection, sample storage, DNA extraction and sequencing method. This section will initially focus on aspects that bias molecular based approaches, namely 16S rRNA gene sequencing and metagenomics, before discussing impacts on culture-based approaches. A detailed discussed of sequencing methodology is outside the scope of the current review, but investigators should consider the latest developments in the technology when designing studies. For instance, longer read lengths can improve de novo genome assembly and detection of structural variants,^24^ while measuring absolute abundances can give a more accurate picture of true microbial changes.^16,25^

**Figure 1.**
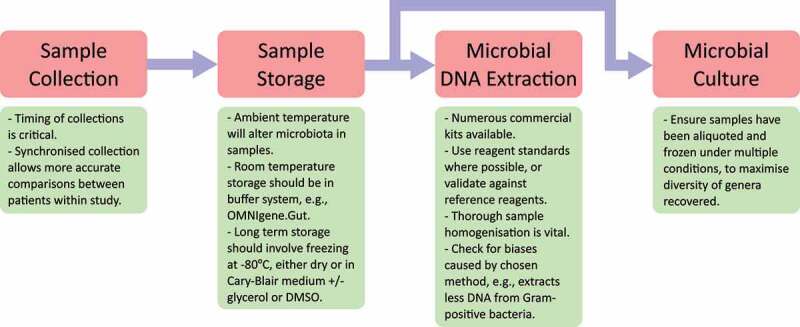
Summary guide for minimizing the introduction of biases when designing and choosing methods for the processing of stool samples for microbiome studies.

The approaches outlined for minimizing the introduction of biases during these stages are summarized in Figure 1.

### i) Sample collection

The composition of an individual’s gut microbiome fluctuates over time, meaning a sample collected on a single given day cannot be said to be a definitive representation of their microbiota. Studies must deploy longitudinal sampling in order to detect, analyze and illustrate this variability. Timing of sample collections is therefore a key factor in any microbiome study.^23^ This will impact comparisons that can be made both between samples within the study and already published datasets. Studying neonates creates challenges to this process as there is no ability to control precisely when a neonate has a bowel movement, they cannot communicate when they are anticipating one, and they are unable to collect their own samples. This process is also affected by whether a neonate is in hospital or at home. Within hospitals, ward staff will make efforts to systematically collect samples, but stool will nevertheless be exposed to ambient temperature and oxygen within the nappy/diaper, often for an undefined period of time. This is an issue as exposure to ambient temperature has been shown to affect the growth or suppression of different bacterial genera within an infant stool sample.^26^ This is in contrast to if a neonate is at home with a parent, who will likely know quite quickly if the neonate has defecated and thus change nappies more rapidly than hospital staff would be able. Parents could then be asked to estimate the amount of time a sample was in a nappy before collection, allowing for a measure of the exposure times to ambient temperatures of samples.

Generally, patients or parents are willing to provide stool samples for research without renumeration. One of the more in-depth analyses of this can be a found in a review evaluating the protocols of the Flemish Gut Flora project, which aimed to survey the adult microbiome.^16^ Of the 3083 individuals who completed all sampling procedures, fewer than 5% reported having thoughts of quitting due to the stool sampling procedure. Of the 1754 individuals who dropped out of the study, only 2.8% reported this was due to needing to store stool samples in their home freezer, while 33% stated it was due to being unable to complete procedures during the timeframe of a sampling round. This demonstrates that aversion to sampling is not a prevalent issue that should dominate the concerns of scientists designing microbiome studies.

### ii) Sample storage

Following collection, samples must be processed and analyzed, or, if this is not immediately possible, put into storage. Proper storage of samples is vital to stabilize the stool microbiome, which has been shown to undergo significant change if left at ambient temperature for 48 hours.^27^ This variation is due to differential growth and death of bacterial species and the degradation of bacterial DNA at such temperatures and oxygen conditions.^28^ If a sample is stored under such conditions, it will not give an accurate measure of the *in vivo* gut microbiome at the time of sampling and will bias subsequent analysis. Sample storage is therefore a key issue for any neonatal microbiome study. This is particularly the case for multi-center studies, or studies which rely on patient samples being collected in the home. For such studies, samples will likely need to be transported to a single research institute for analysis, which lengthens the time before processing commences and increases the chances of microbiota alteration if appropriate collection and storage conditions are not considered.

Multiple studies have been conducted to evaluate the capacity of various storage methods to preserve stool microbiota.^22,26–30^ A recent systematic review of 92 studies found that freezing at −80°C is the most commonly used method, employed in 71.4% of studies.^23^ Storage of neonatal stool samples at this temperature has been confirmed to largely preserve the microbiota for at least 2 years.^31^ In addition, as rapid freezing of samples and transport via a cold chain is not always possible, various storage buffers have also been tested. One of the most widely evaluated has been the commercial OMNIgene.Gut Stool Microbiome Kit (DNA Genotek).^22,26–28^ Choo et al. found that, following 72 hours of storage, refrigeration showed no major divergence in microbiota composition or diversity compared to controls stored at −80°C.^27^ In contrast, samples stored in RNAlater, Tris-EDTA and OMNIgene.Gut showed substantial diversion. Notably, the OMNIgene.Gut kit was the most comparable of the preservation methods tested.

Panek et al. found that samples in the OMNIgene.Gut kit at room temperature for 14 days compared favorably to freezing.^22^ They also highlighted that, compared to freezing, the buffer better preserved Proteobacteria, but could artificially increase the presence of the genus *Sutterella*. A different preservation kit, the Stool Nucleic Acid Collection and Preservation Tube (Norgen BioTek Corp), can also skew the representation of specific genera based on bacterial cell wall structure.^29^ Ten Gram-negative genera had greater abundance in the commercial buffer, while ten Gram-positive genera were more abundant in the frozen samples. Williams et al. focused on ambient temperature storage during postage of infant samples to a lab, comparing OMNIgene.Gut with dry swab and dry sterile tube storage.^26^ They obtained more similar trends to Choo et al., whereby all of their storage methods showed variation from a frozen control, but the commercial buffer showed the least change and was thus deemed suitable for sample collection in study participants’ homes. They also commented on the commercial kit being practical for parents to use, which is likely to maximize sampling response rates.

Such work is focused on how sample storage impacts molecular analyses, in particular 16S rRNA gene sequencing and metagenomic sequencing, and so ultimately reflects the preservation of bacterial DNA. It is also important to consider how direct culture and isolation of bacteria from samples is impacted by these conditions. Culturing viable microbes from stool can identify bacteria that might otherwise be missed with molecular based approaches.^32^ Additionally, isolating microbes allows for further experimental work, including studying the metabolic capacity or microbial-host crosstalk of specific strains.^33^ Biclot et al. compared different cryopreservation conditions, namely dry storage and suspension in Cary Blair transport medium, with or without cryoprotectant glycerol or DMSO.^30^ They found that dry storage and Cary Blair medium with DMSO yielded the highest number of genera. This result for the dry condition supports the use of existing samples stored at −80°C. Another key finding from this study was that cryopreservation conditions differentially impacted the recovery of specific bacterial genera. Ultimately, best practice would be to store stool aliquots in multiple cryopreservation media, to guarantee the widest possible taxonomic spectrum of cultured isolates.

### iii) Extraction and purification of bacterial DNA

Once samples are appropriately collected and stored, microbial DNA must then be extracted and purified prior to downstream molecular work. Before DNA extraction, it is critical that stool samples are thoroughly homogenized. Subsampling of stool has been shown to give highly variable microbiota composition data, attributed to various taxa being encapsulated within different microenvironments, within an individual stool.^34^ Homogenization therefore serves to reduce intra-sample variation. Numerous commercial kits are available that have been optimized for DNA extraction from a range of sample types, including stool. This is reflected in a systematic review of 92 studies focused on the preterm gut microbiome by Westaway et al., who found the choice of extraction method to be highly variable, with 15 different methods used.^23^ The Qiagen QIAmp DNA Stool Kit was the most frequently used (40.3% of studies), followed by using no commercial kit (14.9%) and the Qiagen PowerLyzer PowerSoil Kit (10.4%). The choice of DNA extraction method is an important source of bias in microbiome analysis, based on the homogenization and lysis methods used.^22,23^ Bacterial cell wall structure is a key factor here, as Gram-positive cells have a much thicker peptidoglycan layer and so can require longer or more forceful lysis than Gram-negative cells. Therefore, entire genera could be missed from downstream analysis due to ineffective lysis leaving cell walls intact, or due to overly vigorous lysis degrading the DNA of easily lysed cells.^23^

The biases described above could be addressed through method standardization. Use of an international standardized protocol for microbiome analysis, covering sample collection and storage, DNA extraction, sequencing and bioinformatic pipelines, would reduce confounding factors at each of these stages and make results more consistent.^35–38^ However, such a protocol may not meet the specific needs of a particular research group.^37^ An alternative is to develop a set of reference reagents as international standards, against which other methods can be validated.^38^ Such a system is already used in clinical trials, allowing accurate study-to-study comparisons, and reagent standards can be accredited by existing bodies, such as the World Health Organization. Bioinformatic methods could also be used to normalize data based on the variability introduced by specific methods.^37^

It is vital that researchers make careful and informed choices as to the methods they deploy for sample collection, storage and processing when studying the composition of the gut microbiome. Each method will introduce varying degrees of bias and it is important to make an informed decision on what is most acceptable with consideration for the research questions and hypotheses. For instance, if researchers wish to include existing publicly available data and/or directly compare with previous work, it would be important to mimic the previous methods where possible. A wide range of literature now exists to support such decisions and should be consulted during study design.

## Studying interactions between the neonatal host and the gut microbiome

Descriptive work of the neonatal gut microbiome and how it develops over time remains important and can provide researchers with hypotheses and help to focus subsequent work. Such work includes understanding how the neonatal gut epithelium (i.e., the host) responds and adapts to colonization by bacteria. While it is known that host-microbiome interactions underpin development of both healthy physiology and certain pathologies in infants, detailed mechanisms underlying these processes remain largely unknown.^4,20,39,40^ Attempts to address these research questions are also made difficult by the obvious and important ethical considerations that surround accessing neonatal tissue for research purposes.

The lack of availability of neonatal intestinal tissue for study is a significant barrier to researchers.^41,42^ In adults, gut-related pathologies, such as irritable bowel disease, have been investigated through the use of endoscopic pinch biopsies to obtain tissue for research.^43,44^ However, such procedures are not suitable for neonatal populations and researchers are therefore dependent on using neonatal tissue salvaged from surgical resections (i.e., not collected for research purposes, but using resected tissue that would otherwise be discarded).^45^ Such major surgery is generally performed on infants with severe pathologies, meaning the tissue collected is generally unhealthy. This naturally makes studying normal gut tissue development and function in both preterm and term neonates more challenging, as well as making it difficult to design appropriate control conditions for *ex vivo* experiments aiming to understand gut pathologies.

### i) Animal models

Using animal models with a similar gut anatomy to humans is one solution to this issue. Mouse, rat and pig models for the both the preterm and term neonatal gut have been established.^46–49^ Microbiome research conducted with gnotobiotic mice, rats and pigs has yielded important insights into associations between the gut microbiota and: immune maturation; healthy gut epithelial morphology; skeletal muscle growth; and disease pathology.^46,50–53^ However, these models are still limited in various general aspects, such as the cost of animal care, lack of the genetic variation seen in human populations, inability to fully recreate the complex environmental factors that shape a human gut microbiome and, despite key similarities, differences in anatomy and physiology that prevent a true recreation of human systems.^54,55^ They also have a specific weakness as models of the preterm gut, as preterm mice, rats and pigs are delivered at roughly 90–95% of gestation, whereas a human preterm neonate could be born as early as 60% gestation.^49,56,57^ Therefore, the preterm animal may not accurately model the immaturity of both the immune system and anatomy of the preterm human. Another key drawback relevant to microbiome studies is that these animals possess an endogenous gut microbiome which is different to that of humans.^58^ While methods to generate animals with humanized gut microbiota are well established, it is unclear how well specific human bacterial taxa colonize the guts of these animals and therefore whether the transplanted microbiota function in the same way.^59^

### ii) Ex vivo models

An alternative to animal models is the use of *ex vivo* epithelial cell models, such as Caco-2 cells and intestinal-derived organoids (also referred to as enteroids). Caco-2 cells, originally derived from a human colon carcinoma, can be grown as a polarized monolayer with tight junctions forming between each cell.^60^ For this reason, Caco-2 cells have historically been the principal cell line used as an *in vitro* model for intestinal epithelial enterocytes.^61^ This has included extensive use as a model for the neonatal gut epithelium when investigating the effects of breast milk and members of the microbiota on the neonatal host.^62–65^ However, this cell line is limited in a number of ways. Firstly, Caco-2 monolayers are composed of only one cell type, whereas the intestinal epithelium features multiple, including enterocytes, goblet cells, Paneth cells, stem cells and endocrine cells.^61,66^ They therefore fail to recreate the tissue structure of the gut epithelium. Secondly, the cells are cancer-derived and so will likely have physiological properties that differ from those of normal, healthy epithelial cells.^60^ Finally, the cell line is not patient specific, i.e., is not a primary cell line. This makes studying cellular mechanisms that underpin observable phenotypes in patients far more challenging.

**Figure 2.**
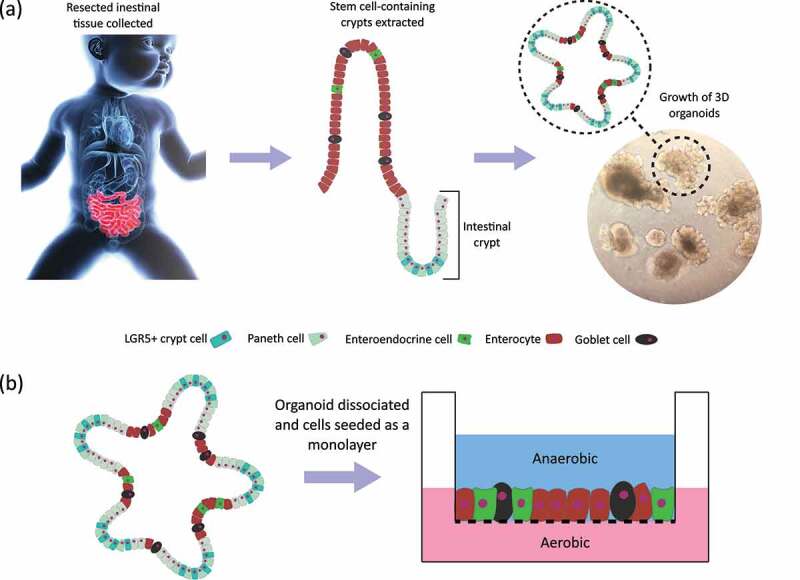
(a) Summary of organoid culture procedure. (b) Schematic of organoid monolayer seeded onto a permeable transwell insert.

Organoid models were developed over the last decade, following the discovery of Lgr5-positive stem cells within intestinal crypts.^67^ Crypts can be extracted from intestinal tissue and the stem cells cultured to grow new intestinal epithelium, as shown in Figure 2(a).^67,68^ Organoids naturally form polarized, 3D-spheroid structures that feature all of the major cell types and are physiologically active tissues. Organoids derived from intestinal crypts can be used to study tissue homeostasis, disease pathology and develop personalized medicine approaches.^69^ They retain the genetic predisposition and immune profile of the host, making them well suited to assays that explore the interactions between the host and the gut microbiome.^70,71^ Importantly for neonatal work, there is evidence to suggest that the life stage of the patient impacts organoid function. For instance, preterm intestinal-derived organoids are different in transcription to adult intestinal-derived organoids.^45^ Strikingly, this difference was most pronounced when studying the organoid response to the presence of live microbes. Such work further supports the utility of intestinal organoids for studying host-microbe interaction and emphasizes the importance of using organoid lines derived from the patient population and life stage of interest.

Alternative cell sources for intestinal organoid culture include induced pluripotent stem cells (iPSCs), embryonic stem cells (ESCs) and intestinal fragments from aborted fetuses.^41,42,72^ One advantage of these cell lines is that they are not dependent on neonatal surgery and therefore also not limited to typically non-healthy tissue. Additionally, differentiation of iPSCs and ESCs simulates embryonic growth and so provides a better model for studying gut development.^69^ But, organoids derived from such cells and fetal-derived organoids lack the link to a patient and their unique phenotypes, such as composition of their gut microbiome and genetic predisposition to disease.

Numerous model systems have been developed to allow host-microbe studies using organoid cell lines, all of which generally involve either microinjection of bacteria into the lumens of 3D organoids, reversing the polarity of 3D organoids, or making a 2D monolayer from organoids.^69^ Microinjection allows the 3D structure of organoids to be maintained and allows for physiologically relevant spatial localization of bacteria within the system (see Figure 2(a)).^73^ Reversing the polarity of 3D organoids involves turning the organoids inside-out, with the basolateral surface in the lumen and the apical surface located on the outer side.^74^ This method bypasses the need for laborious and technically challenging microinjection, as gut microbes and nutrients can simply be added to the surrounding culture medium. Additionally, excess mucus and extruded dead cells produced from the apical surface, which accumulate in the lumen of normal organoids and may confound results, can be easily removed from the culture by refreshing the medium. However, a notable disadvantage of both methods is that neither yet allows for the fine control of oxygen levels within organoid lumens to recapitulate the natural oxygen gradient found across the human intestinal epithelium and maintain viability of anaerobic bacteria.^73,75^

Organoid monolayers are produced by dissociating the spheroidal organoids and seeding the cells onto a surface, the most commonly used being permeable Transwell supports, as illustrated in Figure 2(b).^76^ Tight junctions reform between the cells, producing a polarized, 2D epithelial monolayer. The main advantage of this method is that the Transwell system allows for the basolateral compartment to be sealed off from the apical compartment.^77^ The apical compartment can then be made anaerobic, while the basolateral side remains oxygenated, meaning the natural oxygen gradient across the gut epithelium can be established.^78,79^ This method has been further developed to allow for the continuous perfusion of oxygen into the basolateral compartment, allowing organoids cultures to be run for longer.^78^ Their 2D nature does however mean that monolayer cultures sacrifice the 3D tissue architecture of the gut. Organoid monolayers can also be seeded into microfluidic systems, to create an “intestine-on-a-chip” model.^80^ The fluidic control provided by such systems allows nutrients, metabolites or microbes to be continuously perfused over the epithelial monolayer, under tight spatiotemporal control. These systems can recreate the fluid flows and shear stresses that epithelial cells would usually be subjected to within the intestinal lumen. However, these systems are generally highly labor intensive to set up and complex to use.^78^ Additionally, as with the 3D organoid culture methods, microfluidic systems lack the physiological oxygen gradient of the gut epithelium.

Despite various advantages, organoid models do suffer from limitations. They lack components of the host microenvironment, such as immune cells, neurons and enteric vasculature.^81,82^ Despite the correct cell types being present, organoids cannot form true villus-crypt architecture, meaning the 3D compartmentalization of the gut is not recreated.^83^ Additionally, organoid culture is dependent on matrices, such as Matrigel, and growth factors which are poorly defined, vary from lot to lot and expensive.^69,82^ Furthermore, organoid culture techniques are complex and relatively low throughput.

Nonetheless, in recreating the functional complexity of host tissue, organoids still have a strong advantage over *in vitro* cultures with individual cell types. Co-culture systems which introduce additional cell types, such as immune cells, to organoid cultures, have been developed to increase the complexity and biological relevance of these model tissues.^69^ Using organoids within a co-culture system with bacteria also provides greater flexibility than animal models for studying how different members of the neonatal gut microbiota interact with the intestinal epithelium.^78,84^ Such experiments could allow for the mechanistic role of individual bacterial strains within the neonatal gut microbiota to be elucidated. Collagen scaffolds have also been developed to allow the formation of true villus-crypt architecture within organoids.^83^ Finally, organoids do go some way toward addressing the issue of the lack of availability of neonatal tissue, as culturing stem cells essentially grows more tissue. This allows multiple rounds of experiments to be conducted from a single piece of intestinal tissue and makes research reproducible through the sharing of established organoid cell lines between different groups.

## Conclusion

The gut microbiome plays an important role in neonatal growth and development, impacting health outcomes during both this early stage of life and into adulthood. Studying neonates presents unique methodological challenges and investigators planning on conducting work in this area must appreciate how sample collection, storage and processing can impact results. Study design should ensure cohorts are large enough to include any important covariates and overcome potential confounders, as well as being representative of the wider population of interest. Experimental studies in humans embedded within RCTs will further serve to disentangle potential mechanistic understanding by linking changes in the gut microbiome with efficacy of intervention. Experimental methods must be carefully chosen and standardized where possible, to reduce the introduction of biases when detecting microbiome composition. Representative models of the neonatal gut should also be optimized and used to explore the biological mechanisms underpinning microbiota-mediated effects on the neonatal host. Ultimately, research groups should ensure they share, where they are able, their best practices for overcoming these challenges, to improve the quality of research in this field and help drive it forward as a whole.

## References

[1] Lynch SV, Pedersen O, Phimister EG. The human intestinal microbiome in health and disease. N Engl J Med. 2016.375(24):2369–13. doi:10.1056/NEJMra1600266. 27974040

[2] Gomaa EZ. Human gut microbiota/microbiome in health and diseases: a review. Antonie Van Leeuwenhoek. 2020.113(12):2019–2040. doi:10.1007/s10482-020-01474-7. 33136284

[3] Sommer F, Backhed F. The gut microbiota — masters of host development and physiology. Nat Rev Microbiol. 2013.11(4):227–238. doi:10.1038/nrmicro2974. 23435359

[4] Ahearn-Ford S, Berrington JE, Stewart CJ. Development of the gut microbiome in early life. Exp Physiol. 2022.107(5):415–421. doi:10.1113/EP089919. 35041771 PMC9305283

[5] Battersby C, Santhalingam T, Costeloe K, Modi N. Incidence of neonatal necrotising enterocolitis in high-income countries: a systematic review. Arch Dis Child Fetal Neonatal Ed. 2018.103(2):F182–9. doi:10.1136/archdischild-2017-313880. 29317459

[6] Stewart CJ, Ajami NJ, O’brien JL, Hutchinson DS, Smith DP, Wong MC, Ross MC, Lloyd RE, Doddapaneni H, Metcalf GA, et al. Temporal development of the gut microbiome in early childhood from the TEDDY study. Nature. 2018.562(7728):583–588. doi:10.1038/s41586-018-0617-x. 30356187 PMC6415775

[7] Yatsunenko T, Rey FE, Manary MJ, Trehan I, Dominguez-Bello MG, Contreras M, Magris M, Hidalgo G, Baldassano RN, Anokhin AP, et al. Human gut microbiome viewed across age and geography. Nature. 2012.486(7402):222–227. doi:10.1038/nature11053. 22699611 PMC3376388

[8] Falony G, Joossens M, Vieira-Silva S, Wang J, Darzi Y, Faust K, Kurilshikov A, Bonder MJ, Valles-Colomer M, Vandeputte D, et al. Population-level analysis of gut microbiome variation. Science. 2016.352(6285):560–564. doi:10.1126/science.aad3503. 27126039

[9] Cuna A, George L, Sampath V. Genetic predisposition to necrotizing enterocolitis in premature infants: current knowledge, challenges, and future directions. Semin Fetal Neonatal Med. 2018.23(6):387–393. doi:10.1016/j.siny.2018.08.006. 30292709 PMC6626706

[10] Grech A, Collins CE, Holmes A, Lal R, Duncanson K, Taylor R, Gordon A. Maternal exposures and the infant gut microbiome: a systematic review with meta-analysis. Gut Microbes. 2021.13(1):1–30. doi:10.1080/19490976.2021.1897210. PMC8276657 33978558

[11] Kapourchali FR, Cresci GAM. Early-life gut microbiome—the importance of maternal and infant factors in its establishment. Nutr Clin Pract. 2020.35(3):386–405. doi:10.1002/ncp.10490. 32329544

[12] Vujkovic-Cvijin I, Sklar J, Jiang L, Natarajan L, Knight R, Belkaid Y. Host variables confound gut microbiota studies of human disease. Nature. 2020.587(7834):448–454. doi:10.1038/s41586-020-2881-9. 33149306 PMC7677204

[13] Marian AJ. Molecular genetic studies of complex phenotypes. Transl Res. 2012.159(2):64–79. doi:10.1016/j.trsl.2011.08.001. 22243791 PMC3259530

[14] Casals-Pascual C, Gonzalez A, Vazquez-Baeza Y, Song SJ, Jiang L, Knight R. Microbial diversity in clinical microbiome studies: sample size and statistical power considerations. Gastroenterology. 2020.158(6):1524–1528. doi:10.1053/j.gastro.2019.11.305. 31930986

[15] Ferdous T, Jiang L, Dinu I, Groizeleau J, Kozyrskyj AL, Greenwood CMT, Arrieta M-C. The rise to power of the microbiome: power and sample size calculation for microbiome studies. Mucosal Immunol. 2022.15(6):1060–1070. doi:10.1038/s41385-022-00548-1. 35869146

[16] Vandeputte D, Tito RY, Vanleeuwen R, Falony G, Raes J. Practical considerations for large-scale gut microbiome studies. FEMS Microbiol Rev. 2017.41(Supplement_1):S154–67. doi:10.1093/femsre/fux027. 28830090 PMC7207147

[17] Embleton N, Berrington J, Cummings S, Dorling J, Ewer A, Frau A, Juszczak E, Kirby J, Lamb C, Lanyon C, et al. Lactoferrin impact on gut microbiota in preterm infants with late-onset sepsis or necrotising enterocolitis: the MAGPIE mechanisms of action study. Efficacy Mech Eval. 2021.8(14):1–88. doi:10.3310/eme08140. 34591437

[18] Young G, Berrington JE, Cummings S, Dorling J, Ewer AK, Frau A, Lett L, Probert C, Juszczak E, Kirby J, et al. Mechanisms affecting the gut of preterm infants in enteral feeding trials: a nested cohort within a randomised controlled trial of lactoferrin. Arch Dis Child Fetal Neonatal Ed. pp.F1–8. 2022. doi:10.1136/archdischild-2022-324477 PMC10176413 36396443

[19] Jager KJ, Zoccali C, Macleod A, Dekker FW. Confounding: what it is and how to deal with it. Kidney Int. 2008.73(3):256–260. doi:10.1038/sj.ki.5002650. 17978811

[20] Masi AC, Embleton ND, Lamb CA, Young G, Granger CL, Najera J, Smith DP, Hoffman KL, Petrosino JF, Bode L, et al. Human milk oligosaccharide DSLNT and gut microbiome in preterm infants predicts necrotising enterocolitis. Gut. 2021.70(12):2273–2282. doi:10.1136/gutjnl-2020-322771. 33328245 PMC9231288

[21] Kahlert J, Gribsholt SB, Gammelager H, Dekkers OM, Luta G. Control of confounding in the analysis phase - an overview for clinicians. Clin Epidemiol. 2017.9: 195–204. doi:10.2147/CLEP.S129886. 28408854 PMC5384727

[22] Panek M, Cipcic Paljetak H, Baresic A, Peric M, Matijasic M, Lojkic I, Vranešić Bender D, Krznarić Ž, Verbanac D. Methodology challenges in studying human gut microbiota – effects of collection, storage, DNA extraction and next generation sequencing technologies. Sci Rep. 2018.8(1):5143. doi:10.1038/s41598-018-23296-4. 29572539 PMC5865204

[23] Westaway JAF, Huerlimann R, Miller CM, Kandasamy Y, Norton R, Rudd D. Methods for exploring the faecal microbiome of premature infants: a review. Matern Health Neonatol Perinatol. 2021.7(1):11. doi:10.1186/s40748-021-00131-9. 33685524 PMC7941982

[24] Amarasinghe SL, Su S, Dong X, Zappia L, Ritchie ME, Gouil Q. Opportunities and challenges in long-read sequencing data analysis. Genome Biol. 2020.21(1):30. doi:10.1186/s13059-020-1935-5. 32033565 PMC7006217

[25] Rao C, Coyte KZ, Bainter W, Geha RS, Martin CR, Rakoff-Nahoum S. Multi-kingdom ecological drivers of microbiota assembly in preterm infants. Nature. 2021.591(7851):633–638. doi:10.1038/s41586-021-03241-8. 33627867 PMC7990694

[26] Williams GM, Leary SD, Ajami NJ, Chipper Keating S, Petrosin JF, Hamilton-Shield JP, Gillespie KM. Gut microbiome analysis by post: evaluation of the optimal method to collect stool samples from infants within a national cohort study. PLoS One. 2019.14(6):e0216557. doi:10.1371/journal.pone.0216557. 31188837 PMC6561628

[27] Choo JM, Leong LE, Rogers GB. Sample storage conditions significantly influence faecal microbiome profiles. Sci Rep. 2015.5(1):16350. doi:10.1038/srep16350. 26572876 PMC4648095

[28] Anderson EL, Li W, Klitgord N, Highlander SK, Dayrit M, Seguritan V, Yooseph S, Biggs W, Venter JC, Nelson KE, et al. A robust ambient temperature collection and stabilization strategy: enabling worldwide functional studies of the human microbiome. Sci Rep. 2016.6(1):31731. doi:10.1038/srep31731. 27558918 PMC4997331

[29] Watson EJ, Giles J, Scherer BL, Blatchford P. Human faecal collection methods demonstrate a bias in microbiome composition by cell wall structure. Sci Rep. 2019.9(1):16831. doi:10.1038/s41598-019-53183-5. 31727963 PMC6856092

[30] Biclot A, Huys GRB, Bacigalupe R, D’hoe K, Vandeputte D, Falony G, Tito RY, Raes J. Effect of cryopreservation medium conditions on growth and isolation of gut anaerobes from human faecal samples. Microbiome. 2022.10(1):80. doi:10.1186/s40168-022-01267-2. 35644616 PMC9150342

[31] Shaw AG, Sim K, Powell E, Cornwell E, Cramer T, McClure ZE, Li M-S, Kroll JS. Latitude in sample handling and storage for infant faecal microbiota studies: the elephant in the room? Microbiome. 2016.4(1):40. doi:10.1186/s40168-016-0186-x. 27473284 PMC4967342

[32] Stewart CJ, Marrs EC, Magorrian S, Nelson A, Lanyon C, Perry JD, Embleton ND, Cummings SP, Berrington JE. The preterm gut microbiota: changes associated with necrotizing enterocolitis and infection. Acta Paediatr. 2012.101(11):1121–1127. doi:10.1111/j.1651-2227.2012.02801.x. 22845166

[33] Poyet M, Groussin M, Gibbons SM, Avila-Pacheco J, Jiang X, Kearney SM, Perrotta AR, Berdy B, Zhao S, Lieberman TD, et al. A library of human gut bacterial isolates paired with longitudinal multiomics data enables mechanistic microbiome research. Nat Med. 2019.25(9):1442–1452. doi:10.1038/s41591-019-0559-3. 31477907

[34] Gorzelak MA, Gill SK, Tasnim N, Ahmadi-Vand Z, Jay M, Gibson DL, Heimesaat MM. Methods for improving human gut microbiome data by reducing variability through sample processing and storage of stool. PLoS One. 2015.10(8):e0134802. doi:10.1371/journal.pone.0134802. 26252519 PMC4529225

[35] Szostak N, Szymanek A, Havranek J, Tomela K, Rakoczy M, Samelak-Czajka A, Schmidt M, Figlerowicz M, Majta J, Milanowska-Zabel K, et al. The standardisation of the approach to metagenomic human gut analysis: from sample collection to microbiome profiling. Sci Rep. 2022.12(1):8470. doi:10.1038/s41598-022-12037-3. 35589762 PMC9120454

[36] Warmbrunn MV, Attaye I, Herrema H, Nieuwdorp M. Protocol standardization of microbiome studies—daunting but necessary. Gastroenterology. 2022.162(7):1822–1824. doi:10.1053/j.gastro.2022.03.017. 35283113

[37] Gambardella J, Castellanos V, Santulli G. Standardizing translational microbiome studies and metagenomic analyses. Cardiovasc Res. 2021.117(3):640–642. doi:10.1093/cvr/cvaa175. 32569375 PMC7898946

[38] Amos GCA, Logan A, Anwar S, Fritzsche M, Mate R, Bleazard T, Rijpkema S. Developing standards for the microbiome field. Microbiome. 2020.8(1):98. doi:10.1186/s40168-020-00856-3. 32591016 PMC7320585

[39] Warner BB, Deych E, Zhou Y, Hall-Moore C, Weinstock GM, Sodergren E, Shaikh N, Hoffmann JA, Linneman LA, Hamvas A, et al. Gut bacteria dysbiosis and necrotising enterocolitis in very low birth weight infants: a prospective case-control study. Lancet. 2016.387(10031):1928–1936. doi:10.1016/S0140-6736(16)00081-7. 26969089 PMC5553277

[40] Fan Y, Pedersen O. Gut microbiota in human metabolic health and disease. Nat Rev Microbiol. 2021.19(1):55–71. doi:10.1038/s41579-020-0433-9. 32887946

[41] Hill DR, Huang S, Nagy MS, Yadagiri VK, Fields C, Mukherjee D, Bons B, Dedhia PH, Chin AM, Tsai Y-H, et al. Bacterial colonization stimulates a complex physiological response in the immature human intestinal epithelium. Elife. 2017.6:6. doi:10.7554/eLife.29132. PMC5711377 29110754

[42] Senger S, Ingano L, Freire R, Anselmo A, Zhu W, Sadreyev R, Walker WA, Fasano A. Human fetal-derived enterospheres provide insights on intestinal development and a novel model to study Necrotizing Enterocolitis (NEC). Cell Mol Gastroenterol. 2018.5(4):549–568. doi:10.1016/j.jcmgh.2018.01.014. PMC6009798 29930978

[43] Wu F, Dassopoulos T, Cope L, Maitra A, Brant SR, Harris ML, Bayless TM, Parmigiani G, Chakravarti S. Genome-wide gene expression differences in Crohnʼs disease and ulcerative colitis from endoscopic pinch biopsies: insights into distinctive pathogenesis. Inflamm Bowel Dis. 2007.13(7):807–821. doi:10.1002/ibd.20110. 17262812

[44] Bowcutt R, Malter LB, Chen LA, Wolff MJ, Robertson I, Rifkin DB, Poles M, Cho I, Loke P. Isolation and cytokine analysis of lamina propria lymphocytes from mucosal biopsies of the human colon. J Immunol Methods. 2015.421: 27–35. doi:10.1016/j.jim.2015.02.012. 25769417 PMC4725193

[45] Masi AC, Fofanova TY, Lamb CA, Auchtung JM, Britton RA, Estes MK, Ramani S, Cockell SJ, Coxhead J, Embleton ND, et al. Distinct gene expression profiles between human preterm-derived and adult-derived intestinal organoids exposed to enterococcus faecalis: a pilot study. Gut. 2021.71(10):2141–2143. doi:10.1136/gutjnl-2021-326552. PMC9231289 34921063

[46] Gensollen T, Iyer SS, Kasper DL, Blumberg RS. How colonization by microbiota in early life shapes the immune system. Science. 2016.352(6285):539–544. doi:10.1126/science.aad9378. 27126036 PMC5050524

[47] Zhang Q, Widmer G, Tzipori S. A pig model of the human gastrointestinal tract. Gut Microbes. 2013.4(3):193–200. doi:10.4161/gmic.23867. 23549377 PMC3669164

[48] Walter J, Armet AM, Finlay BB, Shanahan F. Establishing or exaggerating causality for the gut microbiome: lessons from human microbiota-associated rodents. Cell. 2020.180(2):221–232. doi:10.1016/j.cell.2019.12.025. 31978342

[49] Ares GJ, McElroy SJ, Hunter CJ. The science and necessity of using animal models in the study of necrotizing enterocolitis. Semin Pediatr Surg. 2018.27(1):29–33. doi:10.1053/j.sempedsurg.2017.11.006. 29275813 PMC5745061

[50] Lahiri S, Kim H, Garcia-Perez I, Reza MM, Martin KA, Kundu P, Cox LM, Selkrig J, Posma JM, Zhang H, et al. The gut microbiota influences skeletal muscle mass and function in mice. Sci Transl Med. 2019.11(502):eaan5662. doi:10.1126/scitranslmed.aan5662. 31341063 PMC7501733

[51] Kumar A, Vlasova AN, Deblais L, Huang HC, Wijeratne A, Kandasamy S, Fischer DD, Langel SN, Paim FC, Alhamo MA, et al. Impact of nutrition and rotavirus infection on the infant gut microbiota in a humanized pig model. BMC Gastroenterol. 2018.18(1):93. doi:10.1186/s12876-018-0810-2. 29929472 PMC6013989

[52] Isani M, Bell BA, Delaplain PT, Bowling JD, Golden JM, Elizee M, et al. Lactobacillus murinus HF12 colonizes neonatal gut and protects rats from necrotizing enterocolitis. PLoS One. 2018;13:e0196710. 29933378 10.1371/journal.pone.0196710 PMC6014650

[53] Bell RL, Withers GS, Kuypers FA, Stehr W, Bhargava A, Yildirim A. Stress and corticotropin releasing factor (CRF) promote necrotizing enterocolitis in a formula-fed neonatal rat model. PLoS One. 2021.16(6):e0246412. doi:10.1371/journal.pone.0246412. 34111125 PMC8191945

[54] Lu P, Sodhi CP, Jia H, Shaffiey S, Good M, Branca MF, Hackam DJ. Animal models of gastrointestinal and liver diseases. animal models of necrotizing enterocolitis: pathophysiology, translational relevance, and challenges. Am J Physiol Gastrointest Liver Physiol. 2014.306(11):G917–28. doi:10.1152/ajpgi.00422.2013. 24763555 PMC4042110

[55] Nguyen TL, Vieira-Silva S, Liston A, Raes J. How informative is the mouse for human gut microbiota research? Dis Model Mech. 2015.8(1):1–16. doi:10.1242/dmm.017400. 25561744 PMC4283646

[56] McCarthy R, Martin-Fairey C, Sojka DK, Herzog ED, Jungheim ES, Stout MJ, Fay JC, Mahendroo M, Reese J, Herington JL, et al. Mouse models of preterm birth: suggested assessment and reporting guidelines†. Biol Reprod. 2018.99:922–937. doi:10.1093/biolre/ioy109. 29733339 PMC6297318

[57] Grases-Pinto B, Torres-Castro P, Abril-Gil M, Castell M, Rodriguez-Lagunas MJ, Perez-Cano FJ, Franch À. A preterm rat model for immunonutritional studies. Nutrients. 2019.11(5):11. doi:10.3390/nu11050999. PMC6566403 31052461

[58] Gootenberg DB, Turnbaugh PJ. Companion animals symposium: humanized animal models of the microbiome. J Anim Sci. 2011.89(5):1531–1537. doi:10.2527/jas.2010-3371. 20833767

[59] Clavel T, Lagkouvardos I, Blaut M, Stecher B. The mouse gut microbiome revisited: from complex diversity to model ecosystems. Int J Med Microbiol. 2016.306(5):316–327. doi:10.1016/j.ijmm.2016.03.002. 26995267

[60] Barthe L, Woodley J, Houin G. Gastrointestinal absorption of drugs: methods and studies. Fundam Clin Pharmacol. 1999.13(2):154–168. doi:10.1111/j.1472-8206.1999.tb00334.x. 10226759

[61] Lea T.Caco-2 Cell LineCaco-2 Cell Line.In: Verhoeckx K, Cotter P, Lopez-Exposito I, Kleiveland C, Lea T, and Mackie A, et al. editors.Cham (CH), 2015pp. 103–111. doi:10.1007/978-3-319-16104-4.

[62] Mattar AF, Teitelbaum DH, Drongowski RA, Yongyi F, Harmon CM, Coran AG. Probiotics up-regulate MUC-2 mucin gene expression in a Caco-2 cell-culture model. Pediatr Surg Int. 2002.18(7):586–590. doi:10.1007/s00383-002-0855-7. 12471471

[63] Facinelli B, Marini E, Magi G, Zampini L, Santoro L, Catassi C, Monachesi C, Gabrielli O, Coppa GV. Breast milk oligosaccharides: effects of 2′-fucosyllactose and 6′-sialyllactose on the adhesion of escherichia coli and salmonella fyris to Caco-2 cells. J Matern Fetal Neonatal Med. 2019.32(17):2950–2952. doi:10.1080/14767058.2018.1450864. 29562795

[64] Kondrashina A, Brodkorb A, Giblin L. Sodium butyrate converts Caco-2 monolayers into a leaky but healthy intestinal barrier resembling that of a newborn infant. Food Funct. 2021.12(11):5066–5076. doi:10.1039/D1FO00519G. 33960994

[65] Ling X, Linglong P, Weixia D, Hong W, Wang TT. Protective effects of bifidobacterium on intestinal barrier function in lps-induced enterocyte barrier injury of Caco-2 monolayers and in a rat NEC model. PLoS One. 2016.11(8):e0161635. doi:10.1371/journal.pone.0161635. 27551722 PMC4995054

[66] Zhang Y, Huang S, Zhong W, Chen W, Yao B, Wang X. 3D organoids derived from the small intestine: an emerging tool for drug transport research. Acta Pharm Sin B. 2021.11(7):1697–1707. doi:10.1016/j.apsb.2020.12.002. 34386316 PMC8343122

[67] Barker N, van Es JH, Kuipers J, Kujala P, van den Born M, Cozijnsen M, Haegebarth A, Korving J, Begthel H, Peters PJ, et al. Identification of stem cells in small intestine and colon by marker gene Lgr5. Nature. 2007.449(7165):1003–1007. doi:10.1038/nature06196. 17934449

[68] Sato T, Vries RG, Snippert HJ, van de Wetering M, Barker N, Stange DE, van Es JH, Abo A, Kujala P, Peters PJ, et al. Single Lgr5 stem cells build crypt-villus structures in vitro without a mesenchymal niche. Nature. 2009.459(7244):262–265. doi:10.1038/nature07935. 19329995

[69] Kakni P, Truckenmuller R, Habibovic P, Giselbrecht S. Challenges to, and prospects for, reverse engineering the gastrointestinal tract using organoids. Trends Biotechnol. 2022.40(8):932–944. doi:10.1016/j.tibtech.2022.01.006. 35221125

[70] Stewart CJ, Estes MK, Ramani S. Establishing human intestinal enteroid/organoid lines from preterm infant and adult tissue. Methods Mol Biol. 2020;2121:185–198. 32147796 10.1007/978-1-0716-0338-3_16 PMC8094111

[71] VanDussen KL, Marinshaw JM, Shaikh N, Miyoshi H, Moon C, Tarr PI, Ciorba MA, Stappenbeck TS. Development of an enhanced human gastrointestinal epithelial culture system to facilitate patient-based assays. Gut. 2015.64(6):911–920. doi:10.1136/gutjnl-2013-306651. 25007816 PMC4305344

[72] Spence JR, Mayhew CN, Rankin SA, Kuhar MF, Vallance JE, Tolle K, Hoskins EE, Kalinichenko VV, Wells SI, Zorn AM, et al. Directed differentiation of human pluripotent stem cells into intestinal tissue in vitro. Nature. 2011.470(7332):105–109. doi:10.1038/nature09691. 21151107 PMC3033971

[73] Puschhof J, Pleguezuelos-Manzano C, Martinez-Silgado A, Akkerman N, Saftien A, Boot C, de Waal A, Beumer J, Dutta D, Heo I, et al. Intestinal organoid cocultures with microbes. Nat Protoc. 2021.16(10):4633–4649. doi:10.1038/s41596-021-00589-z. 34381208

[74] Co JY, Margalef-Catala M, Li X, Mah AT, Kuo CJ, Monack DM, Amieva MR. Controlling epithelial polarity: a human enteroid model for host-pathogen interactions. Cell Rep. 2019.26(9):2509–20 e4. doi:10.1016/j.celrep.2019.01.108. 30811997 PMC6391775

[75] Zeitouni NE, Chotikatum S, von Kockritz-Blickwede M, Naim HY. The impact of hypoxia on intestinal epithelial cell functions: consequences for invasion by bacterial pathogens. Mol Cell Pediatr. 2016.3(1):14. doi:10.1186/s40348-016-0041-y. 27002817 PMC4803720

[76] Garcia-Rodriguez I, Sridhar A, Pajkrt D, Wolthers KC. Put some guts into it: intestinal organoid models to study viral infection. Viruses. 2020.12(11):1288. doi:10.3390/v12111288. 33187072 PMC7697248

[77] von Martels Jzh, Sadaghian Sadabad M, Bourgonje AR, Blokzijl T, Dijkstra G, Faber KN, von Martels JZH, Harmsen HJM. The role of gut microbiota in health and disease: in vitro modeling of host-microbe interactions at the aerobe-anaerobe interphase of the human gut. Anaerobe. 2017.44: 3–12. doi:10.1016/j.anaerobe.2017.01.001. 28062270

[78] Fofanova TY, Stewart CJ, Auchtung JM, Wilson RL, Britton RA, Grande-Allen KJ, Estes MK, Petrosino JF. A novel human enteroid-anaerobe co-culture system to study microbial-host interaction under physiological hypoxia. bioRxiv. 2019. doi:10.1101/555755.

[79] Ulluwishewa D, Anderson RC, Young W, McNabb WC, van Baarlen P, Moughan PJ, Wells JM, Roy NC. Live faecalibacterium prausnitzii in an apical anaerobic model of the intestinal epithelial barrier. Cell Microbiol. 2015.17(2):226–240. doi:10.1111/cmi.12360. 25224879

[80] Bein A, Shin W, Jalili-Firoozinezhad S, Park MH, Sontheimer-Phelps A, Tovaglieri A, Chalkiadaki A, Kim HJ, Ingber DE. Microfluidic organ-on-a-chip models of human intestine. Cell Mol Gastroenterol. 2018.5(4):659–668. doi:10.1016/j.jcmgh.2017.12.010. PMC5924739 29713674

[81] Kovler ML, Sodhi CP, Hackam DJ. Precision-based modeling approaches for necrotizing enterocolitis. Dis Model Mech. 2020 13 6:10.1242/dmm.044388. PMC7328169 32764156

[82] Ramani S, Crawford SE, Blutt SE, Estes MK. Human organoid cultures: transformative new tools for human virus studies. Curr Opin Virol. 2018.29: 79–86. doi:10.1016/j.coviro.2018.04.001. 29656244 PMC5944856

[83] Wang Y, Gunasekara DB, Reed MI, DiSalvo M, Bultman SJ, Sims CE, Magness ST, Allbritton NL. A microengineered collagen scaffold for generating a polarized crypt-villus architecture of human small intestinal epithelium. Biomaterials. 2017.128: 44–55. doi:10.1016/j.biomaterials.2017.03.005. 28288348 PMC5392043

[84] Blutt SE, Crawford SE, Ramani S, Zou WY, Estes MK. Engineered human gastrointestinal cultures to study the microbiome and infectious diseases. Cell Mol Gastroenterol. 2018.5(3):241–251. doi:10.1016/j.jcmgh.2017.12.001. PMC5904028 29675450

